# Host-specific viral predation network on coral reefs

**DOI:** 10.1093/ismejo/wrae240

**Published:** 2024-12-06

**Authors:** Natascha S Varona, Poppy J Hesketh-Best, Felipe H Coutinho, Alexandra K Stiffler, Bailey A Wallace, Sofia L Garcia, Yun Scholten, Andreas F Haas, Mark Little, Mark Vermeij, Antoni Luque, Cynthia Silveira

**Affiliations:** Department of Biology, University of Miami, Coral Gables, FL 33146, United States; Department of Biology, University of Miami, Coral Gables, FL 33146, United States; Department of Veterinary Population Medicine, University of Minnesota, St. Paul, MN 55108, United States; Department of Marine Biology and Oceanography, Institut de Ciències del Mar (ICM), Consejo Superior de Investigaciones Científicas (CSIC), Passeig Maritìm de la Barceloneta, 37-49, 08003 Barcelona, Catalunya, Spain; Department of Biology, University of Miami, Coral Gables, FL 33146, United States; Department of Biology, University of Miami, Coral Gables, FL 33146, United States; Department of Biology, University of Miami, Coral Gables, FL 33146, United States; Department Marine Microbiology and Biogeochemistry, NIOZ Royal Netherlands Institute for Sea Research, P.O. Box 59, Den Burg 1790 AB, Texel, The Netherlands; Department Marine Microbiology and Biogeochemistry, NIOZ Royal Netherlands Institute for Sea Research, P.O. Box 59, Den Burg 1790 AB, Texel, The Netherlands; Department of Organismal & Evolutionary Biology, Harvard University, 26 Oxford St, Cambridge, MA, 02138, United States; Department of Freshwater and Marine Ecology, Institute for Biodiversity and Ecosystem Dynamics, University of Amsterdam, P.O. Box 94240, 1090 GE Amsterdam, The Netherlands; CARMABI Foundation, P.O. Box 2090, Piscaderabaai z/n, Willemstad, Curaçao; Department of Biology, University of Miami, Coral Gables, FL 33146, United States; Department of Biology, University of Miami, Coral Gables, FL 33146, United States; Department of Marine Biology and Ecology, Rosenstiel School of Marine, Atmospheric, and Earth Science, University of Miami, Miami, FL 33149, United States

**Keywords:** bacteriophage, infection network, lysogeny, MetaHi-C

## Abstract

Viral infections are major modulators of marine microbial community assembly and biogeochemical cycling. In coral reefs, viral lysis controls bacterial overgrowth that is detrimental to coral health. However, methodological limitations have prevented the identification of viral hosts and quantification of their interaction frequencies. Here, we reconstructed an abundance-resolved virus–bacteria interaction network in the oligotrophic coral reef waters of Curaçao by integrating direct microscopy counts with virus-host links obtained from proximity-ligation, prophage integration, and CRISPR spacers. This network of 3013 individual links (97 unique species-level interactions) revealed that the abundance of free viral particles was weakly related to host abundance and viral production, as indicated by the cell-associated virus-to-host ratio (VHR). The viruses with the highest free and cell-associated VHR, interpreted here as highly productive viruses, formed links with intermediate-to-low abundance hosts belonging to *Gammaproteobacteria*, *Bacteroidia*, and *Planctomycetia*. In contrast, low-production viruses interacted with abundant members of *Alphaproteobacteria* and *Gammaproteobacteria* enriched in prophages. These findings highlight the decoupling between viral abundance and production and identify potentially active viruses. We propose that differential decay rates and burst sizes may explain the decoupling between free viral abundance and production and that lysogenic infections play an important role in the ecology of high-abundance hosts.

## Introduction

The mode of interaction between viruses and their hosts depends on ecological and physiological factors such as their frequency of encounters, absorption rates, host growth rates, and community diversity [1, 2]. Lytic infections represent an antagonistic predator–prey dynamic that may be responsible for approximately 20 to 50% of the bacterial mortality in the ocean daily [3]. However, the ability of temperate viruses to infect their host without lysing introduces nuance to the study of viral ecology. These commensal or mutualistic interactions between temperate viruses and their hosts can be highly prevalent [4–6]. Therefore, quantifying the frequencies of the different types of ecological interactions between viruses and their hosts is necessary for an accurate understanding of the role of viral infections on microbiomes.

A major cause of temperate infections is viral coinfection caused by a high frequency of encounters between virus and host, which is dependent on the density and diversity of the microbial community [1, 7]. The main challenge in estimating the frequency of encounters is the lack of information on which viruses infect which species of bacteria [8]. Who infects whom in natural environments is still a central outstanding question in viral ecology. Cultivation-based studies have identified viruses with a narrow host range and a nested infection network structure [9, 10]. However, cultivation biases limit these studies to very few and often rare community members that can grow in laboratory conditions. Single-cell sequencing can identify *in situ* virus-host pairs, but it is still a laborious method with limited coverage [11]. Proximity-ligation, i.e. MetaHi-C, which captures viral genomes inside bacterial cells, can identify hundreds of potential virus-host pairs in a single sample [12–16]. MetaHi-C cross-links DNA molecules in close physical proximity, creating chimeric DNA sequences that can be identified using shotgun sequencing [12, 17]. This method effectively links bacterial DNA with integrated viral DNA (prophages) and detects viral DNA during lytic infection, enabling the capture of both lysogenic and lytic phases of viral replication [13]. Although MetaHi-C has successfully revealed complex interactions and broad host ranges in microbial-dense environments such as the human gut, wastewater, soil, and hydrothermal vent mats [12–15], this technology has not been transferred to low-density, oligotrophic marine environments.

On coral reefs, the top-down pressure of viral predation on bacteria promotes coral reef health by removing bacterial biomass that is detrimental to corals [18]. By contrast, lysogeny has been proposed as a mechanism for the emergence of coral pathogens in this ecosystem [19–21]. Yet, which bacterial taxa are preferentially lysed or lysogenized is unknown. In other marine systems, the relationship between abundance and viral activity has been debated. A recent study in the Northern Pacific showed that despite high abundances, cyanophages infected *Prochlorococcus* at low rates (0.3%–1.6%), and another study in the Sargasso Sea suggested that phages of the most abundant bacteria, like SAR11, may have low lytic activity [22, 23]. Therefore, the reconstruction of a host- and abundance-resolved virus–bacteria infection network in oligotrophic marine environments, including coral reefs, is imperative for understanding the role of viruses in these ecosystems.

Here, we probed different modes of virus–bacteria interactions in the oligotrophic waters of Caribbean coral reefs by combining MetaHi-C proximity-ligation of the prokaryotic cellular fraction with size-fractionated viromes. We integrated these genomic datasets with epifluorescence microscopy-derived direct counts of bacteria and viruses to generate an abundance- and species-resolved virus–bacteria interaction network.

## Materials and methods

### Sampling and metagenome preparation

Eighteen coral reef sites along the southwestern coast of Curaçao were sampled over two weeks in July 2021 between 9:00 and 11:00 a.m. (Table S1 for sampling locations and dates). Shallow waters in Curaçao feature low nutrient concentrations characteristic of tropical oligotrophic reefs [24]. Water samples were collected from the benthic boundary layer (within 10 cm of the coral surface) via SCUBA at 5 to 10 meters depth using sterile bilge pumps attached to 18 L collapsible carboys. The samples were transported within 20 min in the dark at 25°C to the CARMABI Research Station for processing. In the laboratory, 1 ml of raw seawater sample was fixed with paraformaldehyde for epifluorescence microscopy (described in detail in Appendix S1). The remainder of the sample was pre-filtered through an 8 μm filter (Whatman, Milwaukee, USA). Two-liter subsamples were concentrated on a 0.22 μm Sterivex filter (Millipore Sigma, Burlington, USA) for metagenome sequencing of the cellular fraction. A separate 2 L subsample of the pre-filtered seawater was filtered on a 25 mm diameter 0.22 μm Nucleopore flat filter (Whatman, Milwaukee, USA) for MetaHi-C sequencing. All filters were immediately frozen at −80°C and stored until DNA extraction or fixation and enzymatic digestion (for MetaHi-C). DNA was extracted from Sterivex filters using an adapted protocol of the Nucleospin Tissue Kit (Macherey-Nagel, Duren, Germany). Briefly, Sterivex filters were incubated at 55°C with T1 buffer and Proteinase K overnight in a rotisserie oven, followed by a 30-min incubation period at 70°C with B3 solution. The lysate was then extracted from Sterivex filters using 2 ml syringes and the protocol was continued following the manufacturer’s instructions. A 5 L subsample from the 8 μm pre-filtered seawater was used to generate viral community (virome) concentrates by filtration through a 0.45 μm Sterivex filter (Millipore Sigma, Burlington, USA) followed by concentration of the filtrate down to 50 ml using a VivaFlow 100 kDa tangential flow filtration cassette (Sartorius, Goettingen, Germany). Samples were treated with 0.1% chloroform and transported at 4°C until further processing. Viral samples were treated with 10% Polyethylene Glycol 8000 (Thermo Fisher Scientific, Waltham, USA) overnight and centrifuged at 14 000 × *g* for 2 hours. DNA was extracted from the pellet using the Invitrogen Purelink Viral DNA kit (ThermoFisher, Carlsbad, CA, USA) according to the manufacturer’s instructions. One sample, CUR-010, was lost during this process. Total DNA was quantified in a Qubit 2.0 Fluorometer using a high-sensitivity dsDNA kit (Thermo Fisher Scientific, Waltham, USA). DNA library preparation was conducted at AZENTA, Inc. (South Plainfield, NJ, USA) using the NEB NextUltra DNA library kit (New England Biolabs, Ipswich, MA, USA). Libraries were sequenced using a HiSeq 4000 (Illumina, San Diego, CA, USA) with a 2x150 paired-end configuration. All reads from metagenomes and viromes were trimmed and quality filtered with BBDuk under the following conditions*: ktrim = rl, k = 23 mink = 11, hdist = 1, qtrim = rl, trimq = 30, maq = 30, entropy = 0.90* [25] (Table S2). The resulting clean reads were used for contig assembly with SPAdes using the –meta and –only-assembler flags [26]. Filters for six sites were randomly selected for MetaHi-C sequencing. Briefly, The Hi-C library was created using the Phase Genomics ProxiMeta Hi-C v4.0 Kit, where DNA within cells was crosslinked with formaldehyde, digested with Sau3AI and MlucI, and ligated to form chimeric DNA molecules, which were then pulled down with streptavidin beads [12, 17]. Meta Hi-C processing and quality control methods are described in more detail in Appendix 1 (Table S3).

### Viral genome identification

We used a hybrid machine learning and protein similarity approach, VIBRANT, to classify and annotate viral contigs [27]. VIBRANT references HMM profiles from KEGG, Pfam, and VOG databases and assigns v-scores based on the number of significant hits by the HMM-search. Viral contigs were dereplicated using CD-HIT at 95% nucleotide identity (*cd-hit-est -M 20000 -c 0.95 -aS 0.85*) and binned using vRhyme [28, 29]. The quality of vMAGs and unbinned contigs was assessed by CheckV [30]. To allow CheckV to assess vMAGs that contained multiple contigs per bin, the contigs were linked with 200 N spacers using a vRhyme python script (*link_bin_sequences.py*). Genomes larger than 10 kb or classified by CheckV as complete or high quality were included in downstream analyses. For the construction of the Curaçao Viral Database (CVDB), vMAGs were dereplicated using Virathon, which divides viral genomes into viral populations based on 80% shared genes and 95% average nucleotide identity (ANI) [31] (Table S4). We tested if Hi-C linked viruses were different species from known cultivated viruses from ICTV by using 85% coverage and 95% identity in CheckV’s anicalc.py. The fractional (out of total reads) and relative abundances (out of total viral reads) of each viral genome within the metagenome were calculated by accounting for genome length (described in detail in Appendix 1) [32]. The relative abundances were multiplied by microscopy counts assuming that the viral-like particles (VLP) identified through microscopy are predominantly DNA-containing viral particles. Some of these VLPs may also include RNA viruses [33] or other DNA-containing vesicles and large gene transfer agents, albeit at low abundances [34]. Combined with the loss of rare viral and bacterial genomes in metagenomes, microcopy-corrected abundances are possibly overestimated.

### Prokaryotic MAG binning

In addition to the binning of sequence clusters from proximity-ligated samples [17], prokaryotic bins were generated by assembling metagenome reads into contigs sample-by-sample using meta-SPAdes or by co-assembly of all samples using MEGAHIT v1.2.9 [35, 36]. Contigs were binned both as co-assemblies and sample-by-sample using CONCOCT v1.0 and MetaBAT2 v2.12.1 [37, 38]. Bins were refined using the *bin_refinement* module of MetaWRAP v1.3 [39]. The resulting bMAG quality was assessed by CheckM v.1.2.2 [40]. For capturing Hi-C phage-host linkages, bacterial metagenome-assembled genomes (bMAGs) with a > 20% completion and < 10% redundancy were maintained (final median completion of 74.4% with 2.6% redundancy). bMAGs were dereplicated with fastANI v.1.33 using a threshold of 95% identity, k-mer length of 16, and minimum fragment length of 3000 bp [41]. All bMAGs were combined to create a prokaryotic database of Curaçao’s reefs.

### Viral and bacterial taxonomy and genome relatedness

Bacterial MAGs were classified using GTDB-tk v2 [42]. A phylogeny for bMAGs with links to viruses was constructed using Anvio’s genome similarity function in the program pyANI [43]. Viral genome relatedness was accessed using Dice distances between viral protein-encoding genes to construct a neighbor-joining tree using GL-UVAB_v0.6 [44]. For this, phage genomes dereplicated at 95% identity were compared with genome sequences from the Genomic Lineages of Uncultured Viruses of Archaea and Bacteria (GL-UVAB) database (accessed 04-06-2022) and viruses obtained from the NCBI with updated taxonomy by the International Committee on Taxonomy of Viruses (ICTV, accessed 11-03-2022). The modified Dice distances were calculated based on shared homologous proteins and their degree of identity [45]. The Dice method facilitates genome comparisons even when homologous genes are not universally shared. First, we performed an all-versus-all comparison of protein-encoding genes from the CVDB and ICTV sequences using Diamond in sensitive mode with an e-value ≤ 0.01. The distances between genomic sequences are calculated as _Distance_AB = 1 − (2 × (AB) / (AA + BB)), where AB is the summed bitscores of Diamond matches between sequences A and B, and AA and BB are the summed bitscores of self-matches for sequences A and B. Therefore, a lower _Distance_AB value corresponds to both i) more shared homologous proteins and ii) higher identity between proteins of A and B, whereas nonhomologous proteins increase _Distance_AB due to a lack of matching between A and B but valid self-matches for A and B. The resulting Dice distance matrix is then used to construct a phylogenomic tree through the neighbor-joining algorithm, implemented using the Phangorn package in R. The GL-UVAB_v0.6 script was edited with the -m flag for Prodigal to allow gene calling across N-linkages [46] using this method. The resulting tree Newick file was imported into the Interactive Tree of Life v.6 (iTol) and annotated.

### Virus-host pairing

For Hi-C, metagenome deconvolution was performed with ProxiMeta [17], which utilizes a proprietary MCMC-based algorithm based on Hi-C linkages to create putative genome and genome fragment clusters to generate bMAGs and vMAGs. For vMAGs, contigs were first categorized as viral by VIBRANT [27]. For this method, Hi-C reads were aligned to the assembly using BWA-MEM with -5SP options specified [47]. Quality control methods are described in more detail in Appendix 1. In addition to Hi-C, putative prophages and CRISPR spacers were used to identify potential virus-host pairs. To detect putative prophages, CVDB genomes were aligned against bMAGs using BLASTn v.2.5.0. Alignments with ≥95% identity and ≥ 99% coverage of the entire viral contig in a bacterial contig from a bMAG were considered putative prophages (Table S5). Of 42 potential prophages identified with this approach, 37 displayed flanking regions with a median of 18 775 bp. In five instances, viral contigs were nearly identical in length to the corresponding bMAG contigs (identified in Table S5). Three of them were identified as “provirus” by geNomad [48], two by Hi-C linkage, and one aligned with 100% coverage and 99% nucleotide identity to a complete genome of the same species as the host identified here (bMAG v16bin11 and vMAG vRhyme_bin_4628). Putative prophages were classified as “temperate” along with viruses that encoded integrases as classified by VIBRANT [27]. CRISPR spacers were identified from bMAG contigs using MinCED, which uses CRISPR Recognition Tools [49]*.* These CRISPR spacers were compared against the vMAGs using BLASTn v.2.5.0, and the results were filtered using a maximum of two mismatches/gaps, 100% coverage, and a minimum coverage length of 20 nucleotides (Table S6). We also identified CRISPRs from unassembled reads using the CRISPR recognition tool Crass using default parameters [50]. These were matched to vMAGs using the same BLASTn thresholds as above (Appendix 1) (Table S7). We predicted the host of these vMAGs using RaFAH, which offers ~80% accuracy at the class level at default settings [51].

### Gene annotations

Metabolic genes were identified using METABOLIC [52], which incorporates protein annotation from KEGG, TIGRfam, Pfam, custom hidden Markov model (HMM) databases, dbCAN2, and MEROPS. A metabolic module was considered present in a bMAG if the bMAG contained at least 50% or a minimum of three steps of the module. For vMAGs, we removed genomes with contamination (determined by CheckV) > 0 from the metabolic analysis. The remaining vMAGs were required to have at least one step of a module for the module to be considered present. This resulted in 709 vMAGs encoding genes from 56 unique modules (versus 301 in the bMAGs). inStrain v.1.9.0 was used to calculate PN/PS ratios of viral genes identified by Prodigal [53] in generalist vMAGs by mapping reads to vMAGs using Bowtie2 in sensitive mode. To optimize viral annotation with Pharokka, we only used genes that were also identified by PHANOTATE, a gene prediction program optimized for bacteriophages [54, 55]. Genes labeled as AMGs were excluded from the PN/PS analysis.

**Figure 1:**
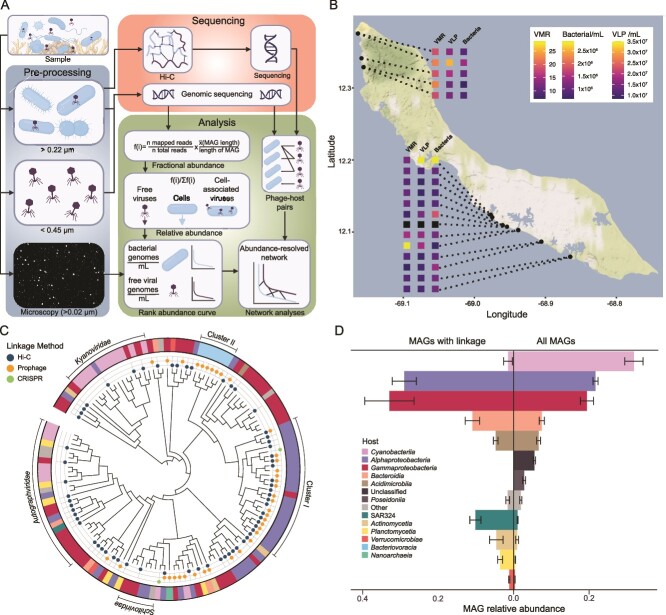
Viral and host abundances, diversity, and interactions. (**A**) a summary of the experimental design of the study, including the four main sample types (cell-associated metagenomes, viromes, Hi-C metagenomes, and microscopy counts) and the downstream abundance calculations and network analyses. (**B**) Sampling sites on the southern coast of Curaçao and associated microscopy counts. The three box columns show microscopy counts for each of the 17 sites (one microscopy sample was lost). The left column shows the virus-to-microbe ratio (VMR), the second column shows the VLP abundance, and the third column shows the bacterial abundance. (**C**) Genomic diversity of viruses identified in this study with links to prokaryotic hosts. The viral proteomic tree was generated based on the all-versus-all distances calculated from protein sequences encoded by CVDB viral genomes (denoted by circles) and their closest relatives among reference viruses recognized by the International Committee for Taxonomy of Viruses (ICTV). Blue dots indicate the presence of a Hi-C linkage, yellow dots indicate a prophage linkage, and green dots indicate a CRISPR linkage. The absence of a dot indicates a reference virus from ICTV. The outer ring color indicates the host identified through links or known hosts for ICTV viruses. Branch lengths were ignored to highlight the tree topology. Clusters I and II indicate clusters of viruses infecting the same host class (with one exception for cluster I). (**D**) Relative abundance of bMAGs (bars indicate the median and the standard error across samples). The left side shows the abundance of bMAGs with a viral linkage, whereas the right side shows the abundance of all identified bMAGs in the dataset.

### Statistics and figures

The bipartite virus–bacteria network was evaluated for nestedness using FALCON with option SS for the null model, where the size and fill of the matrix for the null model are conserved but uniformly randomly shuffled [56]. Modularity was calculated using Newman’s modularity measure with R package Bipartite using the function computeModules() with 100 steps [57]. The bipartite matrix using genome similarity was plotted using pheatmap v1.0.12. To compare modules with genome identities, a neighbor-joining tree of virus genomes was constructed from the Dice distances calculated from the number of shared protein-encoding genes and their similarity in an all-versus-all comparison [45]. bMAG similarity was estimated using ANI and percent genome aligned obtained from Anvio’s function anvi-compute-genome-similarity using the Python program pyANI (−-program pyANI) [58] and the resulting Hadamard-distance matrix. The network with nodes and edges was generated using Cytoscape v3.9.1 [59]. The normality of vMAG and bMAG abundances was assessed using the Shapiro–Wilk test using shapiro.test() from the R package dplyr v1.1.0. If non-normal, a Kruskal-Wallis test followed by a Wilcoxon rank-sum test was used to compare differences in abundance between lytic and temperate viruses with and without linkages, as well as for bacteria. To compare the correlation between virus and host abundances, a Spearman’s rank correlation test was carried out with R package stats v4.2.2. All violin plots and barplots were generated using the R package ggplot2 v3.4.1. The AMG heatmap was generated by using pheatmap v1.0.12.

## Results

### Frequency of virus–host links

To investigate virus-host interactions on coral reefs, we developed an approach combining size-fractionated metagenomes, viromes, and epifluorescence microscopy with proximity ligation (Hi-C) (Fig. 1A). Epifluorescence microscopy revealed a median bacterial density of 9.6 × 10^5^ cells per ml in seawater overlying coral reefs at 18 sites along the southwestern coast of Curaçao (Fig. 1B, Table S1), falling within the second quartile of densities observed in a meta-analysis of 11 ecosystems between the ultraoligotrophic oceanic gyres and coastal waters, and within values observed in oligotrophic coral reefs [60]. Cellular metagenomes (> 0.22 μm) and viromes (< 0.45 μm) generated 1 406 318 856 reads, and MetaHi-C (> 0.22 μm) generated 1 497 536 932 chimeric reads (Tables S2 and S3). Metagenome and virome reads were assembled into 72 294 799 contigs, of which 61 948 were classified as viral and binned into viral metagenome-assembled genomes (vMAGs). Quality control and dereplication resulted in 4019 representative putative viral genomes that comprise the Curaçao Virus Database (CVDB). An all-versus-all comparison of protein-encoding sequences between CVDB and viruses classified by the ICTV showed that all identified CVDB viruses belong to the class *Caudoviricetes*, of which 11 clustered with previously described *Prochlorococcus* and *Synechococcus* phages. Among viruses with Hi-C links, 15 were classified at the family level, belonging to *Autographiviridae*, *Kyanoviridae*, and *Schitoviridae* (Fig. 1C). A comparison between the CVDB and uncultivated viruses from the Genomic Lineages of Uncultured Viruses of *Archaea* and *Bacteria* (GL-UVAB) database [19] showed that 3269 CVDB viruses shared genes with previously identified uncultivated viruses (Fig. S1) but did not cluster at the species level and 750 CVDB viruses did not cluster with previously sequenced genomes at the thresholds applied here.

Putative hosts of CVBD viruses were identified among 292 representative bMAGs binned from metagenomes and Hi-C sequences and dereplicated at the species level (Table S8). Across all samples, 3013 individual virus-host predicted interactions were identified: 1959 Hi-C links, 997 prophage links, and 57 CRISPR links (Table S9). Dereplication of viruses at 80% shared genes and 95% nucleotide identity and hosts at the species level yielded 70 high-confidence, unique Hi-C links, 42 putative prophage links, and two CRISPR links. These links were formed between 88 dereplicated viruses and 51 host bMAGs representing 97 unique predicted pairs (Table S10). Viruses within each clade of the GL-UVAB tree were linked with hosts from distinct classes, with two exceptions: cluster I, containing 23 unclassified viruses, mostly linked to *Alphaproteobacteria* (only one *Gammaproteobacteria* host), of which 18 viruses were linked to hosts of the *Sphingomondales* order (Cluster I, Fig. 1B); and cluster II, consisting of putative prophages exclusively identified in the class *Bacteriovoracia* (Cluster II, Fig. 1B). None of the Hi-C-linked viruses clustered with ICTV viruses at the species level (95% identity and 85% coverage, according to the minimum information about an uncultivated virus genome standard) [61].

When accounting for host abundances, most virus–bacteria links were identified within *Gammaproteobacteria* (mean = 32.9%, Standard Error [herein, SE] = 6.5%), *Alphaproteobacteria* (mean = 29.0%, SE = 3.2%), *Bacteroidia*, *SAR324*, *Actinomycetia*, and *Plactomycetia* (Fig. 1D, left-side bars; Table 1). *Cyanobacteria* comprised 31.2% of the bacterial community in these samples (SE = 2.4%; Fig. 1D, right-side bars), but represented only 1.5% (SE = 1.2%) of the bMAGs with links (Fig. 1D). This contrasted with an overrepresentation of *Alphaproteobacteria* and *SAR324* with virus links relative to their overall abundance in the bacterial community. We tested the effect of quality thresholds on the recovery of links to verify if these thresholds removed viruses with links to high-abundance hosts (Appendix 1). However, the overall distribution of links remained the same (Fig. S2). The frequency of interactions was also qualitatively compared to an independent method, the Lysis Index from Mortality by Ribosomal Sequencing (MoRS; Fig. S3), with similar results. Host prediction with RaFAH (Fig. S4a) also qualitatively agrees with the link frequency distribution shown in Fig. 1D. To determine if the low frequency of *Cyanobacteria*-virus interactions in the Hi-C dataset was due to CRISPR-mediated resistance not initially captured, we searched unassembled reads for spacers matching viruses and discovered that most spacers matched viruses predicted to infect *Cyanobacteria*, suggesting resistance from previous infections (Fig. S4b, Table S6). The MoRS, RaFAH, and CRISPR comparisons are fully described in Appendix 1.

**Table 1:** Species abundances within the *Gammaproteobacteria* and *Alphaproteobacteria* bMAGs with highest contribution to the group of bMAGS with viral links (Fig. 1D).

**Class**	**Species**	**Mean relative abundance (%)**
*Gammaproteobacteria*	Alteromonas sp002729795	16.0
	Pseudoalteromonas sp013349995	2.6
	*Alteromonas macleodii*	0.7
	Pseudomonas sp003205815	0.6
	*Acinetobacter* sp.	0.5
	*Pseudomonas pachastrellae*	0.4
	Unclassified at the species level	3.0
*Alphaproteobacteria*	UBA8309 sp001627655	5.8
	*Qipengyuania flava*	5.3
	*Sphingobium abikonense*	3.6
	AG-430-B22 sp002728335	2.3
	*Brevundimonas sp.*	1.2
	*Sphingobium yanoikuyae*	1.2
	Ponticaulis sp002699325	0.7
	UBA2020 sp002334985	0.5
	Croceicoccus sp002694745	0.5
	M30B80 sp015693905	0.2
	Unclassified at the species level	0.7

### Nested and modular virus–bacteria interaction network

**Figure 2:**
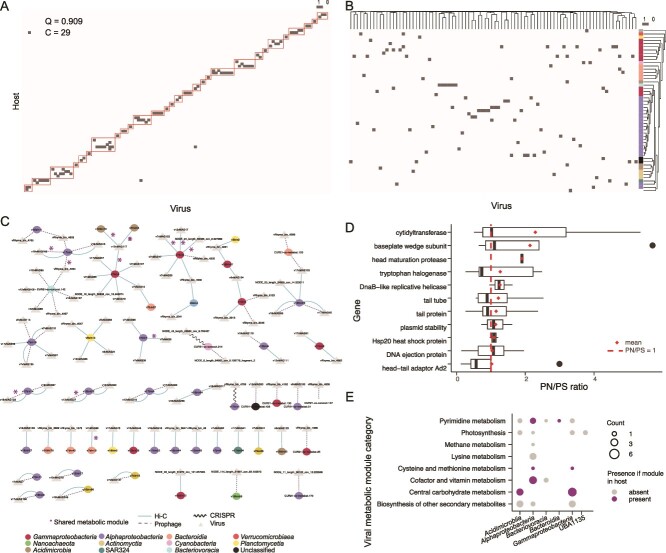
Virus–bacteria interaction network. (**A**) Bipartite network sorted by modularity (normalized modularity score Q = 0.9099; number of clusters C = 29). The dark grey square indicates a virus-host link, and the red squares indicate modules. (**B**) Bipartite virus-host network sorted by genome similarity. Virus similarity was calculated from all-versus-all distances between protein-encoding sequences. The bMAG dendrogram was calculated using average nucleotide identity and percent of genome aligned. (**C**) Visualization of the distribution of link types (line type) across host taxonomy (node color). (**D**) PN/PS ratios for viral genes identified in the generalist viruses in the top left side of panel a. Only genes with an average PN/PS > 1 are shown. (**E**) Metabolic gene functions for viruses with different hosts. Magenta bubbles indicate that genes from the same module (pathway) were identified in both the host and virus.

A bipartite network analysis revealed that the structure of the virus–bacteria interaction network was both significantly modular (Fig. 2A, normalized modularity score (Q) of 0.9099 with 29 modules, where a Q score of 1 indicates perfect modularity) and nested (Nestedness temperature calculator (NTC) = 0.955, *P* value <0.001; nestedness metric based on overlap and decreasing fill (NODF) = 0.7985499 and *P* value <0.001) [57]. The bipartite matrix was sorted by viral and bacterial genome distances to test if modules correspond to genome relatedness (Fig. 2B). The genome-sorted matrix did not reproduce modules, indicating that genetically similar viruses did not systematically interact with genetically similar hosts (Fig. 2B). For instance, 90% of the viruses linked to the group *Alphaproteobacteria* did not share any genes or sequence similarity at the homology thresholds used here. Even among those that did share sequence identity, ANI was 15.5% (SE = 2.9%). Similarly, among viruses infecting *Gammaproteobacteria*, 98.3% did not share genes, and for those with sequence similarity, ANI was 13.0% (SE = 4.69%). The network indicated the predominance of specialist behavior, with 90.9% of viruses interacting with only one host and most hosts interacting with one virus (Fig. S5a and S5b, respectively).

Despite the prevalence of specialist behavior, eight putative generalists were linked to different species, and within these, six were linked to hosts from different classes (Fig. 2C). The most generalist virus, v12vMAG117, was linked with *Acidimicrobia* (phylum *Actinomycetota*) and a *Gammaproteobacteria* (phylum *Pseudomonadota*). To investigate the genomic basis of generalism, we calculated PN/PS ratios (proportion of non-synonymous over the proportion of synonymous polymorphisms) for the genes encoded by the generalist viruses. A PN/PS ratio below 1 indicates stabilizing selection, and above 1 indicates diversification. Forty-nine out of 381 genes in generalist viruses had an average PN/PS ratio above 1, 11 of these with functional annotations (Fig. 2D). Of these, 10 were encoded by v12vMAG117, the most generalist virus in our dataset. The two genes with the highest PN/PS ratios encoded for a cytidyltransferase (mean PN/PS = 2.45, SE = 0.91) and a baseplate wedge subunit (mean PN/PS = 2.29, SE = 1.53). Other genes with high PN/PS encoded for tail tube (mean = 1.12, SE = 0.45) and tail proteins (mean = 1.13, SE = 0.62), albeit with high variance across samples.

To investigate the potential for metabolic expansion or overlap between viruses and putative hosts, we identified KEGG metabolic genes in viruses and bMAGs (Tables S11 and S12, respectively). In the entire dataset, 709 vMAGs encoded metabolic genes in 56 unique KEGG metabolic modules, of which the most prevalent were involved in central carbohydrate metabolism, cofactor and vitamin metabolism, and carbon fixation. Likewise, bMAGs encoded 301 unique modules, of which the most abundant were involved in central carbohydrate metabolism, cofactor and vitamin metabolism, ATP synthesis, and carbon fixation. Of the 56 viral metabolic modules, 55 were also found in bMAGs, except N-glycan biosynthesis. Sixteen viruses with host links encoded 44 metabolic modules (19 unique), and 14 shared metabolic modules with their host (Fig. 2C and E), indicating a high degree of potential metabolic overlap. These included genes involved in pyrimidine metabolism, cysteine and methionine metabolism, cofactor and vitamin metabolism, and central carbohydrate metabolism.

### Abundance-resolved virus–host interactions

**Figure 3:**
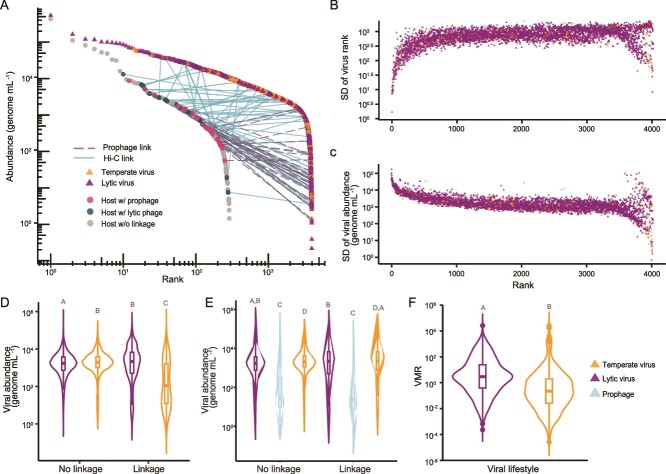
Abundance-resolved network structure. (**A**) Median rank-abundance curve of hosts (circles) and viruses (triangles) across all samples (curves for each sample are displayed in Fig. S6). Solid lines connect Hi-C linkages, and dashed lines connect prophage linkages. (**B**) Variance of the viral rank (indicated by its standard deviation across samples) sorted by the median viral rank. (**C**) Variance of viral abundances (indicated by its standard deviation across samples when accounting for microscopy counts) sorted by the median viral rank. (**d**) Abundances of predicted temperate and lytic viruses with and without linkages. (**E**) Same as (d), but temperate is divided into prophage and temperate. (**F**) Virus-to-host ratio (VHR) of predicted lytic and temperate viruses. Letters above violins indicate groups with significant differences in abundances (Wilcoxon-rank sum test, *P* value <0.05).

As genomic relatedness was not a strong predictor of virus–bacteria links (Fig. 2B), we investigated whether abundances of hosts and viruses were good predictors of interactions by reconstructing an abundance-resolved interaction network (Fig. 3A). The genome length-normalized relative abundances of viruses and hosts were scaled by the total abundance of viral-like particles and cells per ml of sample enumerated by epifluorescence microscopy, generating the estimated number of genome copies of each virus and host per ml of seawater. The median ranks of viruses were not significantly correlated with the median ranks of their linked hosts across samples (Spearman’s rank correlation, *P* value = 0.072, ρ = 0.184). Within sites, four out of the 18 displayed significant positive correlations between virus and host rank (Fig. S6; *P* value <0.05 for CUR21–3, CUR21–11, CUR21–13, CUR21–18 sites). The overall weak relationship between host and viral ranks was analyzed through variance in viral ranks and abundances. Top-ranking viruses displayed low standard deviation in rank (Fig. 3B), showing that these viruses remained dominant across sampling sites. However, these top-ranking viruses displayed large standard deviation in abundances across samples (Fig. 3C), which was reflected in a higher variance in the virus-to-host ratio (VHR; Fig. S7). Site-level relationships are shown in Appendix 1 (Fig. S8).

We further investigated the relationship between viral abundance and lifestyles. Viruses were classified as temperate if their genome encoded integrases or if the genome was identified as a potential prophage integrated in a bacterial MAG. Temperate viruses were significantly more abundant than lytic viruses among viruses without Hi-C linkages (Wilcoxon-rank sum test *P* value <2.2e-16; Fig. 3D). Viruses with Hi-C linkages showed the opposite trend, with lytic viruses displaying higher abundance than temperate viruses (Wilcoxon-rank sum test *P* value <2.2e-16). When the temperate category is broken down into temperate phages (those with integrase but not observed integrated) and putatively integrated prophages, prophages displayed the lowest abundances (Fig. 3E), demonstrating the role of integrated prophage sequences in bringing down the abundances of temperate viruses with links in Fig. 3D. The median VHR for each predicted virus-host pair was lower for temperate viruses versus lytic viruses (Fig. 3F; Wilcoxon *P* value <2.2e-16). bMAGs with a Hi-C link to either a lytic or temperate virus had higher abundances than those without a linkage (Wilcoxon-rank sum test *P* value = 7.999e-10), demonstrating the expected bias of Hi-C for identifying linkages in abundant bMAGs that have higher sequencing coverage leading to high-confidence predictions [12].

The 10 most abundant bMAGs, classified as *Prochlorococcus* sp.*, Canditatus Pelagibacter*, *Synechococcus* sp.*, Candidatus Actinomarina*, and unclassified *SAR86* and *SAR324,* did not have viral links (Fig. 3A). Likewise, the 10 most abundant viruses did not display host links. Their high variance in abundance (Fig. 3C) could have interfered with the detection of links, but lowering Hi-C thresholds for link detection only identified one additional link in this group, between the second-ranked phage and four hosts classified as *Cyanobacteria*, *Gammaproteobacteria*, *Alphaproteobacteria*, and *Bacteroidia* (Appendix 1, Fig. S2, Fig. S9). Host prediction based on sequence identity suggested that these viruses potentially infect *Gammaproteobacteria*, *Cyanobacteria*, and *Alphaproteobacteria* classes with representatives among the top 10 most abundant bMAGs without links (Table S13). It was, however, not possible to link these viruses to hosts at the species level.

### Differences in abundances between free- and host-associated viruses

**Figure 4:**
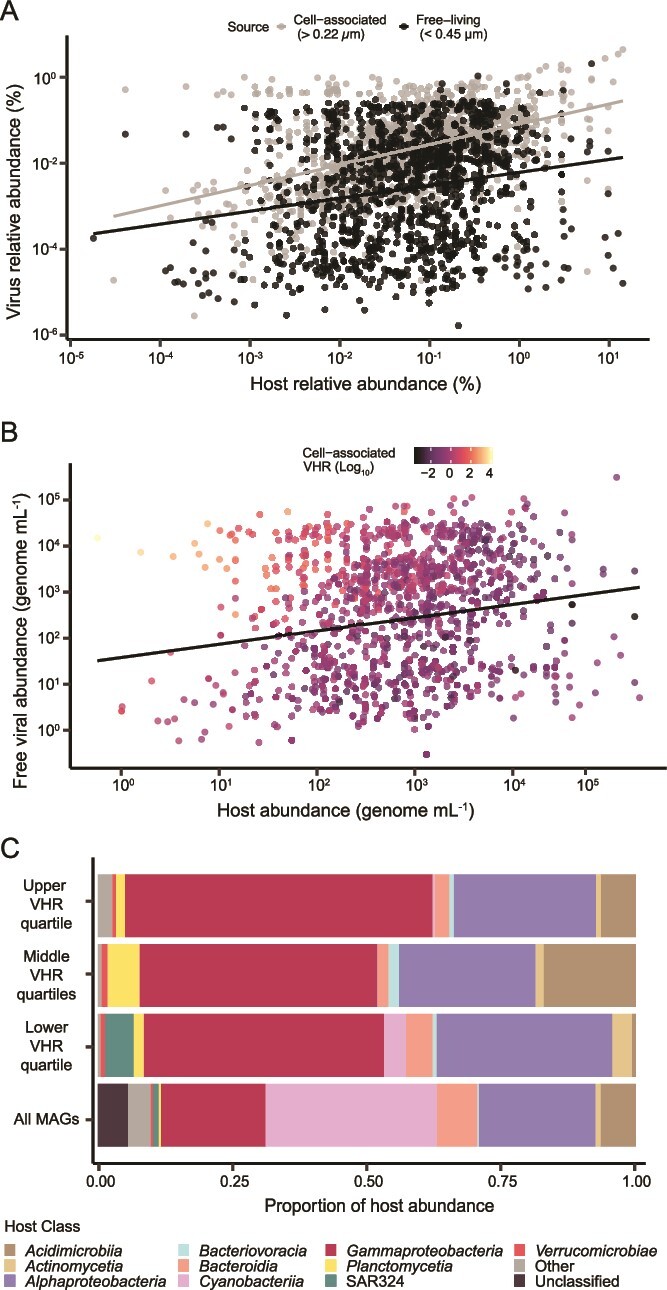
Relationships between viral and host abundances. (**A**) Relationship between host and viral relative abundances in the cell-associated fraction (> 0.22 μm, light gray symbols, linear regression, *R*^2^ = 0.21 and *P* value <0.001;) and free fraction (< 0.45 μm, black symbols, linear regression, *R*^2^ = 0.02 and *P* value <0.001). (**B**) Relationship between host and free viral abundances (< 0.45 μm) in genomes per ml when direct counts from microscopy are incorporated (linear regression, *P* value = 1.014e-12, *R*^2^ = 0.03), and informed by the cell-associated VHR (linear regression between viral abundance and cell-associated VHR is shown in Supplementary Fig. 9, *P* value <2e-16, *R*^2^ = 0.08). (**C**) Relative abundances of hosts interacting with viruses in each VHR quartile compared with the relative abundances of all bMAGs in the dataset.

Host relative abundances poorly predicted viral relative abundances (linear regression between cell-associated viruses and host abundances *R*^2^ = 0.21, *P* value <0.001; and linear regression between free viruses and host abundances *R*^2^ = 0.02, *P* value <0.001; Fig. 4A). Adding microscopy counts slightly strengthened this relationship for free viruses (*R^2^* = 0.03, *P* value = 1.014e-12), but the association remained weak. Free viral abundance had a weak but significant association with cell-associated VHR (*R*^2^ = 0.08, *P* value <2e-16), which was driven by a few high-abundance viruses (Fig. 4B and Fig. S10).

Because cell-associated viral abundances were more strongly associated with host abundances, the cell-associated fraction likely captured ongoing infections at the time of sampling. The cell-associated VHR was, therefore, used as a proxy for viral production or activity, following Marbouty et al., 2021. This assumes that high viral production may result from multiple mechanisms, including large burst sizes, high infection rates, or short infection cycles. Using this interpretation, linked prophages can only achieve high VHR if a large fraction of the host population encodes that prophage, whereas in the case of lytic infection, many copies of the viral genome are made, so even a smaller fraction of infected host cells can yield a high frequency of links. The cell-associated VHR negatively correlated with host abundance (*R*^2^ = 0.23, *P* value <2e-16). Upper quartiles of cell-associated VHR comprised viruses linked to *Gammaproteobacteria*, *Alphaproteobacteria*, and *Acidimicrobia* hosts, indicating skewed viral production within these groups. The medium VHR category included *Acidimicrobia* and *Planctomycetia* links, and the low production group was enriched in *Actinomycetia* and *SAR324* links. Despite being overrepresented among linked hosts, *SAR324* and *Planctomycetia* showed low cell-associated VHR (Fig. 4C).

**Figure 5:**
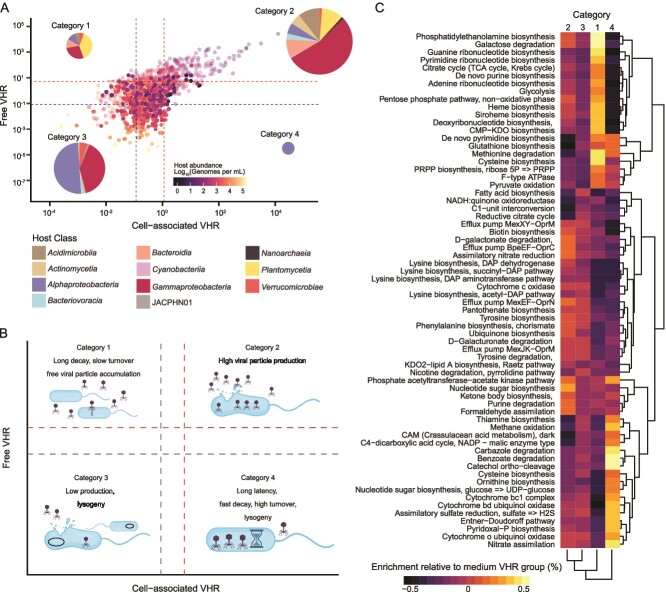
Virus-to-host ratios in free and cell-associated fractions. (**A**) Relationship between the free and cell-associated VHRs (linear regression, *P* value <2e-16, *R*^2^ = 0.52, slope = 0.48). The solid symbols indicate predicted pairs where the bMAG carries a prophage and transparent symbols indicate prophage absence. The red lines represent the upper VHR quartiles, and the blue lines represent the lower quartiles. These quartiles divided the predicted virus–host pairs into four categories, starting from top left: 1) pairs with low cell-associated VHR and high free VHR (n = 18); 2) pairs with high VHR in both cell-associated free fractions (n = 170); 3) pairs with low VHR in both the cell-associated and free fractions (n = 96); and 4) pairs with high cell-associated VHR and low free VHR (n = 6). Host taxonomy in each category is shown in the pie charts. (**B**) Conceptual figure displaying potential mechanisms driving each category: 1) a low cell-associated VHR with a high VHR in the free-living fraction could be caused by low viral particle turnover rates, where particles are slow to decay in the environment relative to their production rates, leading to a high free VHR relative to the cell-associated. 2) High VHR in both fractions could be caused by highly productive viruses due to large burst sizes, short infection times, or a high proportion of infected host cells. 3) Low VHR in both fractions could be caused by overall low viral production, with the opposite underlying mechanisms compared to the high productivity group just described. 4) High VHR in the cell-associated fraction but low VHR in the free fraction may result from high viral particle turnover or long latent periods. (**C**) Metabolic modules enriched and depleted in bMAGs of each VHR category, displayed as the difference in abundance compared to the frequency in bMAGs in the middle VHR values.

High VHR in both free and cell-associated fractions was interpreted here as successful viral replication with host cell lysis and release of free viral particles. VHR had a significant relationship between both fractions (linear regression *R*^2^ = 0.51, *P* value <2e-16; Fig. 5A), and allowed the classification of predicted virus–host pairs into four categories based on VHR quartiles and their possible interpretations (Fig. 5B): category 1 contained virus-host pairs with low cell-associated VHR and high free VHR, and was dominated by viruses linked to *Planctomycetia* and bMAGs enriched in cysteine and methionine metabolism, indicating a role of these pairs in sulfur cycling. Most pairs (n = 170) fell into the category 2, a group with high VHR in both fractions (high-production group), which was mostly linked to intermediary abundance hosts belonging to *Gammaproteobacteria* and lower abundance hosts in the *Bacteroidia*, *Acidimicrobia*, and *Planctomycetes*. This group mostly contained lytic viruses and likely have high viral particle production (Fig. 5B) These hosts were enriched in genes for energy acquisition and nitrogen cycling (Fig. 5C). Category 3 (low VHR in both fractions, indicating low viral production and host mortality, n = 96) was dominated by links to *Alphaproteobacteria*. Among category 3 hosts (low production), 61% had potential prophages, compared to 8% in category 2 (high production). Category 4 (high cell-associated VHR and low free VHR, n = 6) was mainly comprised of *Sphingobium yanoikuyae* bMAGs (n = 4) enriched in catechol and benzoate degradation, C4 carbon fixation, and nitrate assimilation genes.

## Discussion

Understanding viral impacts on microbiomes hinges on identifying infection networks, which has been limited to cultured bacteria-phage pairs or low-resolution computational predictions. Here, we present an *in situ,* species-level, and abundance-resolved virus-host interaction network in oligotrophic coral reefs by integrating microscopy, proximity ligation, and viromics. This network revealed that host abundance poorly predicted free viral abundances, suggesting a decoupling between virion abundances and production. Productive viruses, indicated by a high VHR in both free and cell-associated fractions, interact with intermediately to low abundant hosts belonging to *Gammaproteobacteria*, *Planctomycetes*, and *Bacteroides*. Whether the viruses target low-abundant hosts or if the low abundance results from top-down control through viral predation cannot be ascertained here. These findings emphasize the need for host- and abundance-informed data for the study of viral roles in marine microbiomes.

### Lytic viral infections may benefit coral reefs

*Gammaproteobacteria* and *Alphaproteobacteria* encompassed most bMAGs with virus links (Fig. 1D). Low-abundance groups such as *SAR324* and *Bacteroidia* were overrepresented in the group with links compared to their abundance in the overall dataset. These results were corroborated by MoRS, an independent method to estimate taxon-specific viral lysis (Fig. S3), and are consistent with a previous study in coastal seawater showing that copiotrophic and low-abundance bacteria, including *Gammaproteobacteria* and *Bacteroidetes,* were more frequently targeted by viral lysis [62]*.* On coral reefs, preferential top-down control on copiotrophic *Gammaproteobacteria* could benefit corals because these microbes consume large amounts of oxygen and include opportunistic pathogens that are detrimental to the reef, which may explain large-scale associations between virus-to-microbe ratios and coral cover in the Pacific Ocean [18].

*Cyanobacteria* were the most abundant bMAGs in our data, but were underrepresented among bMAGs with viral links. One explanation for this observation is a possible bias of the proximity-ligation method where host DNA degradation by T4-like cyanophages would prevent the detection of chimeric sequences [63]. However, T4-like phages linked to *Gammaproteobacteria* and *Alphaproteobacteria* groups were abundant in our dataset (*Kyanoviridae* group in Fig. 1B). Another methodological bias could come from the difficulty in assembling cyanobacterial MAGs [64, 65]. Yet, cyanobacterial MAGs with the same completion as the mean across the dataset were assembled here, comprising ~30% of the community (Fig. 1D; Table S4). In fact, Hi-C performed better with highly abundant genomes (Wilcoxon rank-sum test comparing abundances of hosts with and without Hi-C links, *P* value <0.05), helping resolve complex and difficult-to-assemble genomes due to the physical links, ruling out the possibility the assembly biases are responsible for the cyanobacterial skew [12]. Cyanobacteria-phage links were underrepresented even in low threshold tests and in the independent assembly-free MoRS analyses (Appendix 1, Fig. S2, Fig. S3). As an alternative explanation, cyanobacteria may be more resistant to phage infection, which is supported by unassembled read-based CRISPR spacer mining showing an overrepresentation of *Cyanobacteria* as predicted host (Fig. S4a). The low frequency (0.3%–1.6%) of cyanophage infection is consistent with that reported in coastal and open-ocean waters using independent methods, even where both *Cyanobacteria* and cyanophage were abundant [22, 62]. In the subtropical North Pacific, the overall infection rate for the cyanobacterial population averaged less than 1% despite high cyanophage and *Prochlorococcus* abundances, with only a subset of this oceanographic transect displaying high infection frequency [66]. It is still possible that we missed cyanophage infections due to sampling during morning hours, as cyanophage activity has been observed to peak during sunset hours [67–69]. Additional time series studies will be necessary to address this question.

### Phage-bacteria networks are both modular and nested

Marine viruses and bacteria isolated on agar plates have displayed large-scale modularity and local nestedness within each module [9, 10], showing that infections occur preferentially within modules containing strains of the same species. Here, in an oligotrophic coral reef environment, the network was modular and nested, in broad agreement with cultivation studies [9, 10]. Most viruses were specialists and only infected one host at the species level (Fig. 2A). Nested-modular interaction networks have been proposed to be produced by the host–phage coevolution modes in which infection depends on genetic matching between viral tail fibers and host receptors as a result of the predator–prey arms race [70]. Here, genome similarity between species was not a strong predictor of modules, indicating the nestedness may be limited to very closely related strains within the same species (Fig. 2B). A study of deep-sea microbial mats using proximity-ligation showed widespread generalism, with viruses capable of interacting with hosts of entirely different classes or domains, i.e. *Archaea* and *Bacteria* [15]. Whereas specialism was dominant in our dataset, we also identified eight potentially generalist viruses, six of them interacting with different host classes, including one putatively infecting three hosts in different phyla, *Actinomycetota* (class *Acidimicrobiia*), and *Pseudomonadota* (class *Gammaproteobacteria*) (Fig. 2C). Although rare, phages capable of infecting different classes [71] and species [72] have been isolated. The most generalist virus observed here exhibited a positive selection of several genes, including a predicted baseplate wedge subunit directly involved in initiating host infection [73]. Diversification in this gene could allow infection even when tail fiber binding is not ideal, expanding the host range. The difference between the dominant reef specialism versus deep sea mat generalism could be explained by unique host metabolisms and ecological roles [74, 75]. Generalism could be selected in high-density environments where hosts are in close proximity due to syntrophic interactions [15]. In oligotrophic waters, low host densities and high diversity may also select for host range expansion and generalism [76]. Mathematical models and experimental tests with a synthetic community show that, given the fitness cost for generalism, specialists dominate when hosts are engaged in cross-feeding [77]. This scenario aligns with our results, given that syntrophism is common in oligotrophic marine environments [78]. Alternatively, the differences between specialism and generalism between studies could be accounted for by non-productive infections due to post-injection resistance mechanisms and other forms of viral genome transfer between cells [79, 80]. However, low-frequency phage-host interactions of this type would likely have been removed from our dataset due to stringent false positive removal in the Hi-C pipeline (Supplementary Data Methods*;* Quality control of MetaHi-C)*.*

Most metabolic modules identified in viral genomes were also present in their hosts (Fig. 2D), indicating a large degree of metabolic overlap for generalist and specialist viruses. Genes associated with carbon, vitamin, pyrimidine, cysteine, and methionine metabolism were the most common. Viruses likely use these genes to rewire host energy metabolism toward viral particle production during infection [81–83]. The only amino acid metabolism genes shared with hosts were associated with cysteine and methionine metabolism, which are implicated in the sulfur relay system. Sulfur cycling is a critical process in coral reef biogeochemistry [84, 85]. The bacterial degradation of dimethylsulfoniopropionate produced by symbiotic algae that live inside coral tissues has been postulated to act as a nutrient source for corals and help structure bacterial communities of the coral holobiont [86]. The prevalence of sulfur metabolism genes in the viral community suggests that viral infection is an important component of sulfur cycling in this ecosystem.

### Decoupling between free viral abundance and production

The cell-associated VHR was used as a proxy for viral productivity [13], assuming that more productive viruses have more viral genome copies per host genome copy. Mechanisms generating this pattern may include large burst sizes, high absorption rates, short infection times, or a large fraction of infected host populations [87, 88]. The decoupling between free viral abundance and production can be inferred from 1) the lack of correlation between virus and host ranks (Fig. 3A), 2) the weak relationship between free virus abundance and host abundance (Fig. 4A), which was slightly improved with microscopy counts (Fig. 4B), and 3) the stronger relationship between cell-associated viral abundance and host abundance, presumably coming from hosts undergoing infection (Fig. 4A). The cell-associated VHR was weakly related to free viral abundance (Fig. S10), which helps to explain the lack of links for the most abundant viruses by suggesting low lytic activity within this group (Fig. 3A). Alternatively, a recent lysis event that has killed all or the majority of their hosts could cause this pattern. However, this is an unlikely scenario because the most abundant viruses maintain the highest rank across all sample sites over the course of the two sampling weeks while changing in relative abundance (Fig. 3B and C, respectively). The lack of links could also be explained by viral capsid stability and long decay rates, resulting in viral particles remaining in the environment long after production and without incurring new infections [87]. A decoupling between free and cell-associated viral abundances has been previously observed in other oligotrophic marine systems [23]. Together, these results suggest an unrecognized role of viral decay in the abundance and composition of marine viral communities, but further studies are required to test this hypothesis.

From the strong positive relationship between VHRs in free and cell-associated fractions, four categories of virus links could be identified (Fig. 5A). Category 1 had few members, which displayed high free and low cell-associated VHR (n = 18). This could be a result of stable viral particles with slow decay rates that accumulate in the environment over time even with low frequency of infections, or could be caused by extremely large burst sizes [7,89]. In resource-limited conditions, viruses with low multiplication rates may invest in higher capsid stability rather than progeny size [90]. The most predominant host in this category was *Planctomycetia*, one of the groups with the highest mortality in the MoRS lysis index, in alignment with the link data (Fig. S3). *Planctomycetia* typically exhibits slow growth rates with doubling times from days to weeks, which may lower viral multiplication rates, allowing for increased capsid stability [91]. Category 4, with low free and high cell-associated VHR, was also rare (n = 6). This pattern of VHR requires either fast decay rates relative to production, high infection frequencies with small burst sizes, or small fractions of infected hosts with large burst sizes [7,73,92]. This category may also include viruses that exist predominantly as prophages [93]. Four of the six viruses in category 4 interacted with *S. yanoikuyae,* a marine bacterium adapted to low-nutrient environments known to degrade toxic aromatic compounds [94]. This host may serve as a target to investigate vulnerability to lysogeny and long-term interactions.

Categories 2 and 3, comprising the high and low ends of both free and cell-associated VHRs, contain most putative virus-host links. Category 2, with high free and cell-associated VHR, included most links (n = 170), presumably involving productive infections with large burst sizes, short incubation periods, and high encounter frequencies. This group was enriched in low-abundance hosts in the copiotroph groups *Gammaproteobacteria*, *Planctomycetia*, *Bacteroidia*, and *Acidimicrobia* relative to their overall representation in the dataset (Fig. 5A). These results indicate that most viral production occurs among hosts with low to intermediary abundances, in agreement with data from a viral mortality metric using free ribosomes and observations from coral holobionts [62,95]. If true, the viruses in this category are implicated in most of the lysis-mediated biogeochemical cycling in this environment [62,96]. Kill-the-Winner dynamics would predict higher lytic rates for the host with the highest growth rate [97]. It is hard to disentangle the effects of abundance and growth rates in our dataset, but the observed pattern of productive infections occurring on less abundant hosts in Category 2 may be counter to Kill-the-Winner dynamics. The low host abundance could instead result from lytic top-down control, but that is not strongly supported by the weak relationship between free virus abundance and host abundance [90]. The low viral productivity category 3, with low free and cell-associated VHR, contained high-abundance hosts enriched in prophages, aligning with the Piggyback-the-Winner model of increased lysogeny at high densities [1, 4]. Alternatively, the high abundance of lysogens may be a result of prophages conferring protection from lytic infection through superinfection exclusion, which fits the Make-the-Winner framework [1]. Further research into the growth rates of individual hosts and viral decay is required to test these models more accurately.

In conclusion, our study introduces an abundance-resolved virus–bacteria interaction network from an oligotrophic coral reef environment, revealing hundreds of previously unknown interactions. Free viral abundance was a poor predictor of VHR, an indicator of the relative viral production. Instead, high VHR was observed among hosts with intermediary and low abundances. These predicted virus-host-pairs presumably exert a proportionally larger effect on biogeochemical cycling and community dynamics through lysis. These findings also imply that mechanisms including slow turnover rates (slow decay) are likely important for maintaining the abundance of top-ranked viruses. The lowest VHRs in the dataset were observed among intermediary to high abundance hosts, most of which contained putative prophages. These results indicate the importance of lysogeny among abundant coral reef bacteria.

## Supplementary Material

Supplementary_Data_wrae240

Supplementary_Tables_wrae240

## Data Availability

Raw sequence reads are available through the NCBI Sequence Read Archive (SRA) under PRJNA975592. Viral and bacterial genome sequences (MAGs and contigs) and CRISPR spacers are available on Figshare under DOI 10.6084/m9.figshare.23313773, DOI 10.6084/m9.figshare.23306036, and 10.6084/m9.figshare.25504711.v1, respectively.

## Code availability

Bash scripts used to process sequence data are available via GitHub (https://github.com/Silveira-Lab/Varona-et-al-2023-Phage-host-network-structures).
