# Supplementary material for: Host-specific viral predation network on coral reefs
Source: ISME J. 2024 Dec 6;18(1):wrae240. doi: 10.1093/ismejo/wrae240 (PMC11694666; doi:10.1093/ismejo/wrae240)
Supplement: Supplementary_Data_wrae240 [file supplementary_data_wrae240.docx]

**Appendix 1**

**Host-specific viral predation network on coral reefs**

Natascha S. Varona^1^, Poppy J. Hesketh-Best^1,2^, Felipe H. Coutinho^3^, Alexandra Stiffler^1^, Bailey A. Wallace^1^, Sofia L. Garcia^1^, Yun Scholten^4^, Andreas F. Haas^4^, Mark Little^5^, Mark Vermeij^6,7^, Antoni Luque^1^, Cynthia Silveira^1,8*^

**Supplementary Methods**

*Epifluorescence microscopy*

One mL of seawater and 66 µL of 36% PFA were filtered onto 0.02 μm Anodiscs (Cytiva, USA). Anodiscs were stored flat at -20℃ for transportation from Curaçao to the University of Miami, FL. Anodiscs containing bacterial and viral-like particles (VLP) were stained with 100 μl of 10X (v./v.) SYBR Gold Nucleic Acid Gel Stain (Invitrogen, USA) and dried in the dark for 30 minutes. Excess stain was washed off by wetting the Anodisc from the bottom up with two rounds of 100 μl milli-Q water. The bottom side of the filter was gently wiped across Kimtech Science™ Kimwipes™, before being placed on a glass microscope slide with 10 μl of mounting solution (100 µl of 10% ascorbic acid, 4.9 mL of 1X phosphate-buffered saline, and 5 mL of 100% glycerol, 0.02 μm filtered), and a glass slide mounted with another 10 μl mounting solution. Slides were stored at -20℃ until visualized using a ZEISS Axio Imager.A2 equipped with an Axiocam 506 mono camera and the X-Cite Mini (Excelitas Technologies, USA). A single sample was lost (CUR-008), therefore lacking microscopy counts for the respective metagenome. Slides were visualized under oil immersion at 630X magnification, and images were taken using the Zeiss software Zen, 10 images per sample were taken to serve as technical replicates. Cells and VLPs were size delimited using Zen and counted manually. The mean number of cells and VLPs per image was scaled to the total number of fields of view per Anodisc to yield the number of VLPs and cells per milliliter for each sample.

*Genome-length normalized relative abundance calculations and integration of microscopy counts*

The abundance of each viral genome was calculated by mapping QC-ed reads to viral genomes at 95% identity with SMALT v.0.7.6. at 95% identity (*GitHub - Rcallahan/Smalt: SMALT Sequence Aligner*, n.d.). When a read mapped to two contigs at the same identity, one was chosen at random. The number of reads mapped to each contig (*r(i)*) was extracted using samtools (H. Li et al., 2009). The fractional abundance (*f(i)*) of each viral genome within the metagenome was calculated as follows (Equation 1)(Cobián Güemes et al., 2016):

$Equation 1: f(i) = \frac{r(i)}{T(j)} \times\frac{L(mean)}{L(i)}$

Where *L(mean)* represents the mean viral genome length in the dataset (28,623 bp), *L(i)* represents the length of the viral genome, and (*T(j)*) represents the total number of reads in each metagenome. To estimate the number of viral genomes per milliliter of seawater, *f(i)* was divided by the sum of all *f(i)* within the same metagenome or virome and multiplied by the total number of viral particles enumerated from epifluorescence microscopy (Equation 2). The same approach was used to calculate bacterial abundance:

$Equation 2: Genomes/ml of seawater= \frac{f(i)}{\Sigma f(i)}✕ No. of viral particles/mL.$

Virus-to-Host-ratio (VHR) was either calculated using relative viral abundance ($\frac{f(i)}{\Sigma f(i)})$over relative host abundance ($\frac{f(i)}{\Sigma f(i)})$, or with viral $Genomes/ml of seawater$ over bacterial $Genomes/ml of seawater$. Note that viral-like particle counts can only be used for free VHR calculations, as these counts represent free viruses in the water column. We compared free VHR across viral ranking across sites. Most pairs were present in all sites with varying abundances (Fig. S2).

*Quality control of MetaHi-C*

To generate phage-host pairs, six of the eighteen water samples were collected on 0.2 µm flat filters (Whatman, Milwaukee, USA) and flash frozen at -80 ºC for proximity ligation library prep using the ProxiMeta^TM^ Hi-C v4.0 Kit (Phase Genomics, Seattle, USA) according to manufacturer-provided protocols. Intact cells collected on flat filters were crosslinked using a formaldehyde solution and simultaneously digested with restriction enzymes Sau3AI and MlucI. DNA fragments were proximity ligated with biotinylated nucleotides to create chimeric junctions between sequences within physical proximity *in vivo*. Biotinylated DNA was purified using streptavidin beads and processed into an Illumina-compatible sequencing library. Hi-C libraries were sequenced on an Illumina Novaseq, generating 160M 2x150 paired-end reads. Simultaneously, to create a sample-specific reference library an aliquot of the original DNA sample was extracted with a ZYMObiomics DNA miniprep kit (Irvine, CA, USA) and prepared using ProxiMeta library preparation reagents for 100M 2x150 paired-end read sequencing, with an Illumina Novaseq. MetaHi-C and metagenomic libraries were uploaded to the Phase Genomics cloud-based bioinformatics portal for subsequent analysis.

Metagenomic samples were filtered, trimmed for quality, normalized using fastp with default parameters, and then assembled with MEGAHIT using default options (D. Li et al., 2015, 2016). Only contigs larger than 1000 bp were kept for analysis. Duplicates were flagged using SAMBLASTER and discarded from the downstream analysis (Faust & Hall, 2014). Alignments were filtered using samtools using the -F 2304 flag to filter out non-primary and secondary alignments (H. Li et al., 2009). Metagenome deconvolution was performed with ProxiMeta^TM^ (Press et al., 2017), which utilizes a proprietary MCMC-based algorithm based on Hi-C linkages to create putative genome and genome fragment clusters to generate bMAGs and vMAGs. For vMAGs, contigs were first categorized as viral by VIBRANT (Kieft et al., 2020). For this method, Hi-C reads were aligned to the assembly using BWA-MEM with -5SP options specified (H. Li & Durbin, 2010). The first step in identifying links was to compute the normalized connectivity ratio, the density of Hi-C links per kb^2^ of sequence between the virus and the bMAG compared to the connectivity of the bMAG to itself and normalized to the estimated copy count (Uritskiy et al., 2021). A two-step process was used to determine the connectivity ratio. Read counts connecting each pair of clusters were normalized by accounting for the estimated abundance for each contig (Equation 3).

$$Equation 3: C=\frac{V}{H}\frac{L}{\sum L\left( v \right)}$$

The average copy count (C) is the average viral copy count per cell, which is equal to the difference in viral abundance (V) over host abundance (H), times the number of links (L) over the total links between the virus and all other possible hosts. Next, a normalized connectivity ratio is calculated to determine if the linkage density is similar to what would be expected by random chance (Equation 4).

$$Equation 4:R^{'}=\frac{D_{VH}}{CD_{H}}$$

The normalized connectivity ratio (R’) is equal to the ratio of the connectivity density (links per kb^2^) between virus and host (D_VH_) and the average copy count times the connectivity density of the host genome to itself (D_H_). To be considered a successful linkage, R’ needs to be greater than 0.1. Additionally, at least 2 Hi-C reads links had to be detected per virus-host pair per sample and at least 10 links of intra-MAG connectivity to remove false positives. This is done to determine if the connectivity density is similar to what would be expected by random chance if this was the correct host. To determine the optimal cut-off value for the minimum copy count, a receiver operating characteristic (ROC) curve was constructed by plotting the decline in the number of prokaryotic-viral interactions and the number of viruses with at least one host (Fig. S9). Finally, each vMAG was evaluated for the fraction of host MAGs that it still had connections with to identify “sticky” sequences with a likely high proportion of false positives (i.e., links to too many hosts). These were corrected by removing linkages with an average copy count less than 80% of the highest copy count value for the given viral sequence. To test the effects of these thresholds on the number and diversity of links identified, the last two threshold values were removed, and the data was reanalyzed (Fig. S2). Due to the likelihood of false negatives, we maintained all threshold values for all further analyses.

*Mortality by Ribosomal Sequencing (MoRS) and lysis index calculations*

For the investigation of taxon-specific bacterial mortality, we applied the MoRS approach developed by Zhong et al. 2022 to two Cyanobacteria-dominated samples collected from two of the same sites studied by Hi-C but collected one year later. The MoRS protocol was followed with the following modifications: 1 Liter of seawater was filtered through a 0.22 µm Sterivex, the filtrate containing free ribosomes was concentrated in two steps using 30kDa Vivaflows and 30kDa Amicon filters flash frozen and stored at -80 °C. Ribosomal RNA was extracted from this sample and the Sterivex, and the 16S gene was sequenced from both fractions using the same method as Zhong et al. 2023. For qc and adapter trimming we used bbduk (ktrim=rl k=23 mink=11 hdist=1 qtrim=rl trimq=20, ref=bbmap/resources/adapters.fa, ref=MORS_primers.fa, ref=nextera.fa.gz). Taxonomy was assigned using the updated SILVA v138 database by training ASV features with the prebuilt Naïve Bayes classifier. The lysis index was calculated as described by the MoRS method:

$$Equation 5: Lysis index= \frac{Relative abundance of extracellular rRNA of a taxon}{Relative abundance of cellular rRNA of a taxon}$$

For comparison with the linked bMAG results, a lysis index was calculated as:

$$Equation 6: HiC Lysis Index= \frac{Relative Abundance of total bMAG community}{Relative Abundance of bMAGs with viral link}$$

*Verification of the low frequency of cyanobacterial links*

Whether the low representation of Cyanobacterial links was due to biases in the Hi-C method was investigated with three approaches: an analysis of Hi-C link detection, the application of an independent method based on a lysis index, and the comparison with bioinformatic host prediction. For the first approach, we removed two steps for link identification: the minimum connectivity ratio and the copy number (see Methods), which are expected to remove low-frequency links. This increased link detection to 3490 links (Fig. S2a), increasing Cyanobacterial links from 0 to 124 and showing that the method is capable of capturing these interactions. However, the overall representation of Cyanobacteria within bMAGs with links remained low relative to other taxa (Fig. S2b). Second, we verified the low frequency of Cyanophage links with the Mortality by Ribosomal Sequencing (MoRS) method (Zhong et al., 2022) on two sites reef dominated by Cyanobacteria (Fig. S3a). This method calculates a lysis index from the ratio of free rRNA (a proxy for recently lysed host) to cellular rRNA via 16S sequencing, as described above. To compare the links detected here with MoRS results, we created a lysis index using the ratio between the abundances of bMAGs with links and total bMAGs. MoRS results qualitatively agreed with the data from links, where, relative to their abundances, Planctomycetia, SAR324, Gammaproteobacteria, and Verrucomicrobia were preferentially lysed, while Cyanobacteria ranked 8^th^ in the lysis index despite their dominance in the bacterial community (Fig. S3b). Finally, we compared the hosts detected using bMAG links with those predicted from genome comparisons with phages with known hosts using bioinformatic prediction using RaFAH, which offers approximately 80% accuracy at the class level at default settings (Coutinho et al., 2021) (Table S1). Again, this approach qualitatively agreed with the two methods described above, showing that most viruses were predicted to infect Gammaproteobacteria and Alphaproteobacteria, with a smaller fraction predicted to infect Cyanobacteria both in the complete CVDB dataset and the subset of viral genomes with Hi-C links (Fig. S4a and b, respectively). To test if the low frequency of Cyanobacteria-virus interactions in the Hi-C dataset could be explained by CRISPR-mediated resistance not captured in our initial analysis due to difficulties in assembling full CRISPR arrays, we searched unassembled reads for spacers that could be matched to a virus. This search revealed that most unassembled spacers matched viruses predicted to infect Cyanobacteria based on sequence similarity with viruses with known hosts (Fig. S4c, Supplementary Table 6), indicating a potential role of resistance due to previous infections.

Links detected with the lower Hi-C threshold are likely to include false positives (Uritskiy et al., 2021) from “sticky” viruses or infrequent links due to phage injection of the genome into a cell without successful replication and eventual sharing of the viral genome during bacterial conjugation (Hwang et al., 2023). Therefore, we kept the more stringent thresholds for further analyses in an attempt to capture high-confidence associations that likely represent true infections.

**Supplementary Figures**


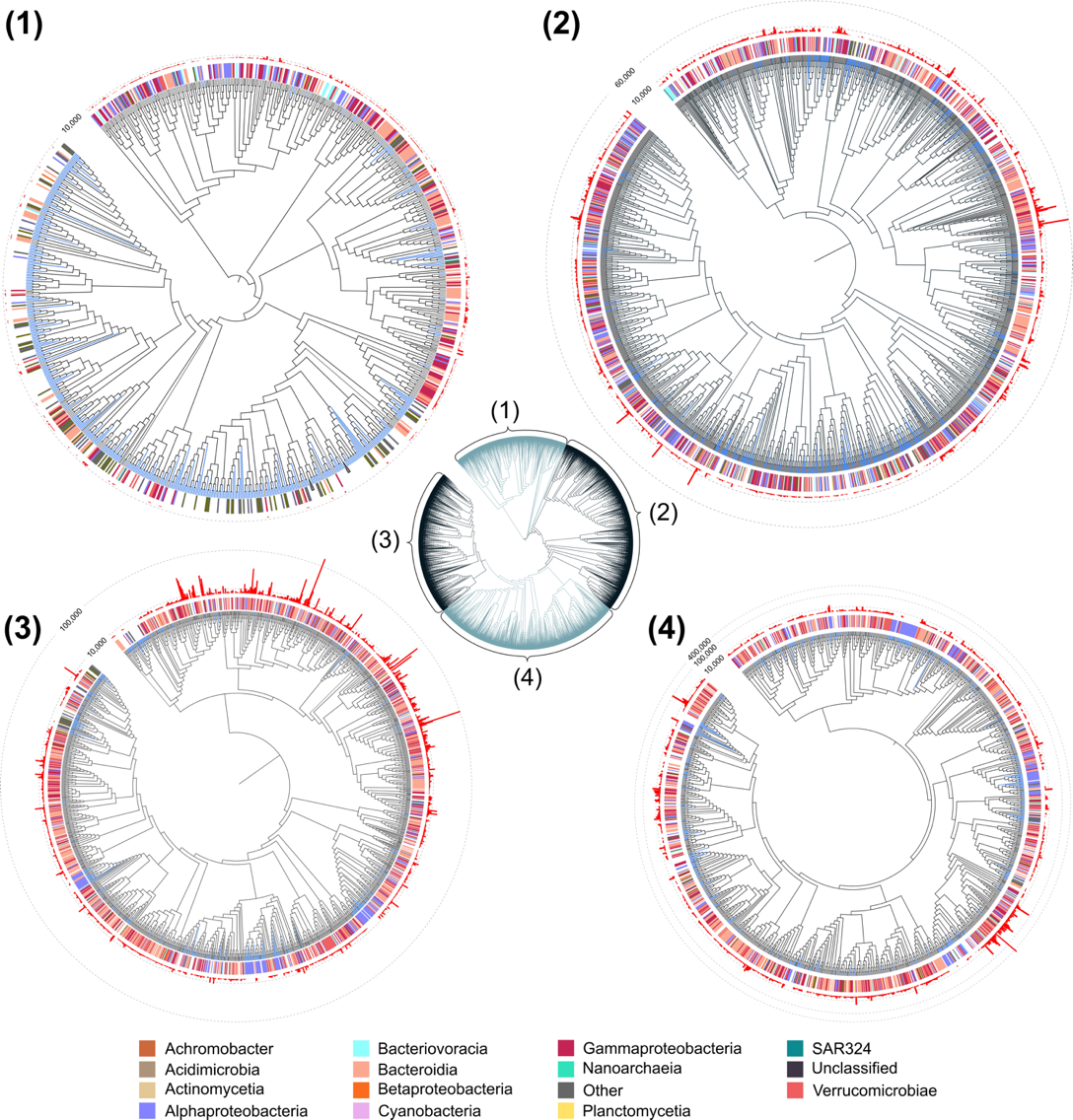


**Supplementary Figure 1. Relationship between viruses sequenced in this study (CVBD) and uncultivated marine and aquatic viruses obtained from the Genomic Lineages of Uncultured Viruses of Archaea and Bacteria (GL-UVAB) database.** The center tree includes all 4019 CVDB viruses and 1548 RefSeq viruses from marine environments. The tree was divided into 4 groups to better display the topology of the tree **(a-d)**. Blue branches represent a GL-UVAB virus, and black represents viruses found in this study. The color ring shows the associated host. The red bar plots represent the median viral abundance in genomes per mL. No CVDB viruses clustered at the species level (> 95% ANI) with reference or GL-UVAB sequences. Distances were calculated based on nucleotide identity and the proportion of shared genes following Coutinho et al. 2019.

**Supplementary Figure 2. Abundances of viruses and hosts with lower Hi-C thresholds for link identification.** The lower threshold was established by removing the connectivity ratio cutoff and the minimum copy number cutoff and is expected to include more false positives. The only requirements for a successful linkage here are that each virus-host pair contains at least 2 Hi-C read links and at least 10 intra-MAG links, yielding 3560 Hi-C linkages. **(a)** Rank-abundance curve of viruses and hosts with decreased Hi-C thresholds, showing higher generalist behavior and new links in higher ranking viruses. **(b)** Relative abundance of MAGs with links (left) and total MAGs (right) using the lower thresholds.

**Supplementary Figure 3. Lytic viral predation as quantified by the Mortality by ribosomal sequencing (MoRS) compared to Hi-C links. (a)** Bacterial relative abundances in the two sites analyzed using MoRS using 16S rRNA sequencing, showing a dominance of Cyanobacteria. **(b)** Lysis indexes, normalized by sample. A Hi-C lysis index was calculated following the MoRS method as the ratio of bMAGs with Hi-C links (representing infected bMAGs) over the total bMAGs.

**Supplementary Figure 4. Host prediction using sequence identity and distribution of CRISPR spacers in unassembled reads.** The software RaFAH was used to predict hosts at the class level for **(a)** all viruses in the CVDB and **(b)** viruses with Hi-C links, showing agreement between the two sets of genomes and an underrepresentation of Cyanobacterial hosts predicted. **(c)** RaFAH-predicted hosts for viruses with matches to 2383 spacers CRISPR spacers in unassembled metagenomic reads, displaying a dominance of Cyanobacteria.


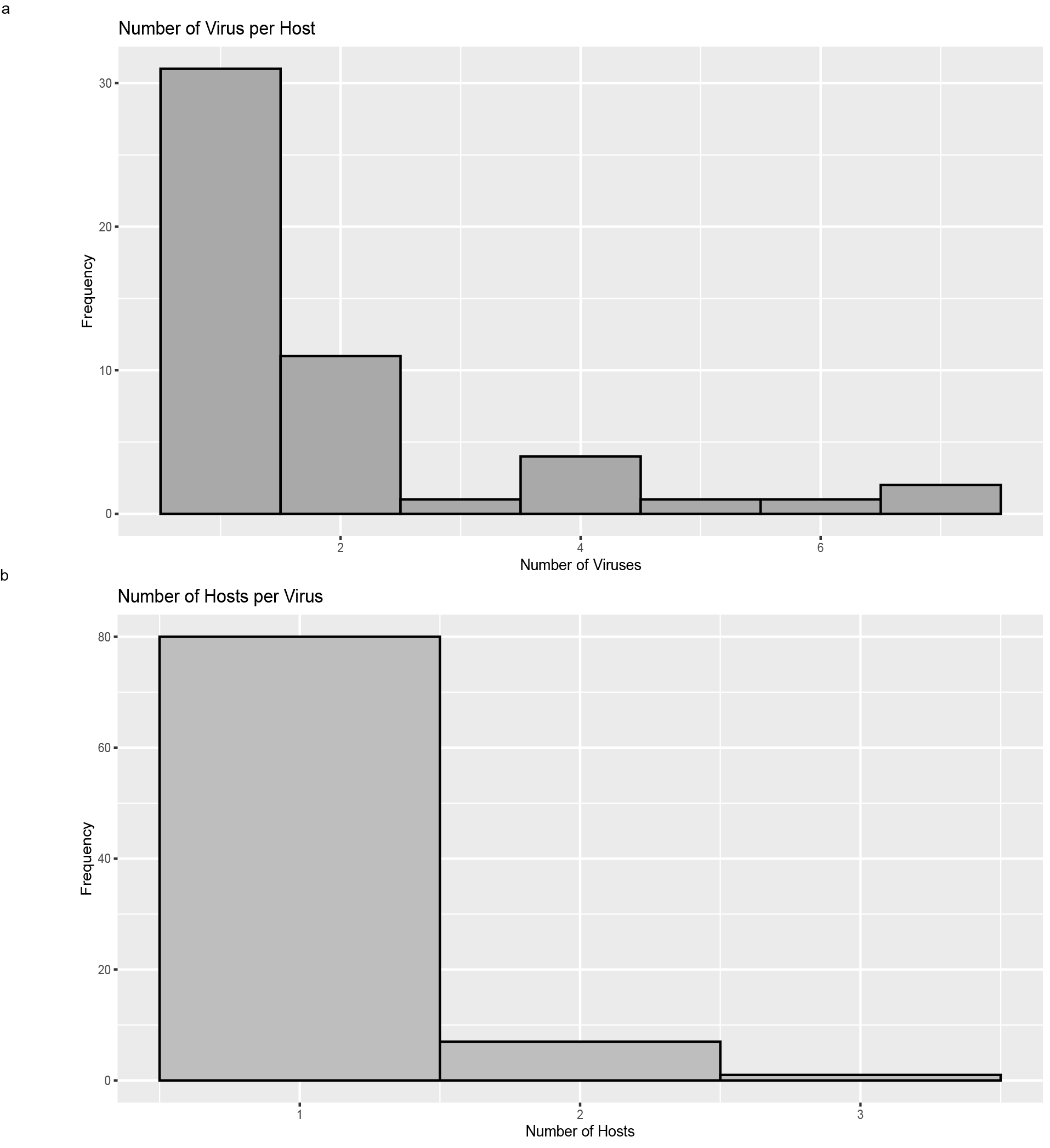


**Supplementary Figure 5. Viral generalism and specialism. (a)** Number of viruses with links to an individual host. **(b)** Number of hosts linked to an individual virus.

**Supplementary Figure 6. Rank-abundance curves for each site.** Bacteria (bMAGs) are indicated by the circles, and viruses by triangles. The x-axis is the abundance of the bMAG and the y-axis is the abundance of the vMAG. Blue lines indicate Hi-C linkages and dashed purple lines indicate prophage linkages. CRISPR linkages were omitted.

**
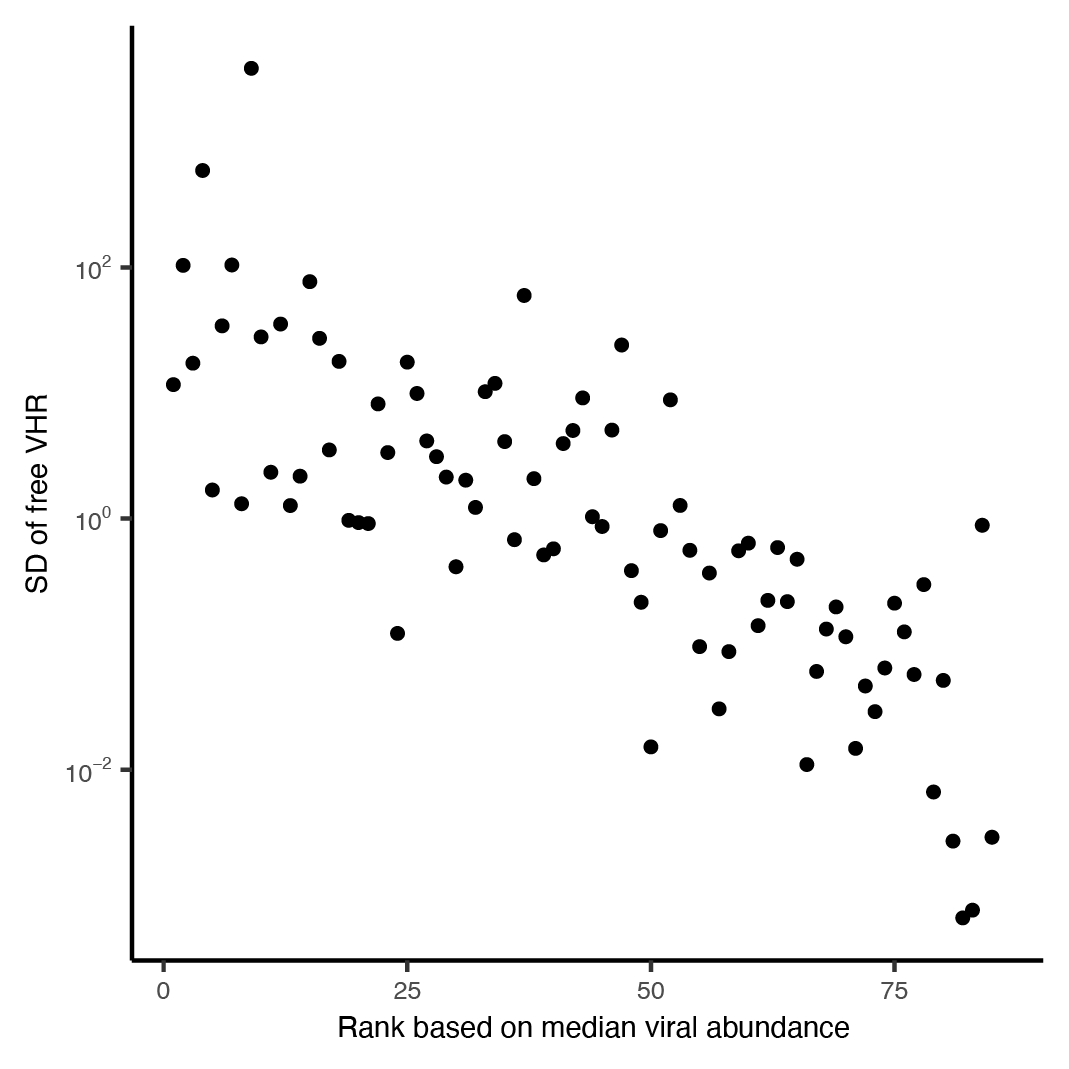
**

**Supplementary Figure 7. Relationship between the variance in cell-associated VHR and viral rank for linked viruses and hosts.** The y-axis displays the standard deviation of VHR across samples, and the x-axis displays the median viral rank. Linear regression p < 2.2e-16 slope= 0.76749 R^2^= 0.07859.

**Supplementary Figure 8. Relationship between cell-associated viral and bacterial relative abundances in each site.** Grey symbols indicate the whole dataset and black indicates links in that specific site. The dotted lines indicate the upper and lower quartiles of cell-associated VHR. Lines falling above the upper dotted line have a high VHR, and the reverse below the lower dotted line. One-way ANOVA showed significant differences in VHR across samples (p-value < 0.001), with sites eight out of the 17 sites with viromes displaying mean VHR above or below the mean of the entire dataset: Sites CUR21-3, CUR21-11, CUR21-13, CUR21-18 (Tukey’s multiple comparisons of mean, p-value < 0.05.

**Supplementary Figure 9. Receiver operating characteristic (ROC) curves for each proximity-ligated sample used for quality control (a-f).** The ROC is generated by plotting the fraction of prokaryotic-viral interactions (x-axis) against the number of viruses with at least one host (y-axis). Linkages with an average copy count of less than 80% of the highest copy count value for the given viral sequence are removed.

**
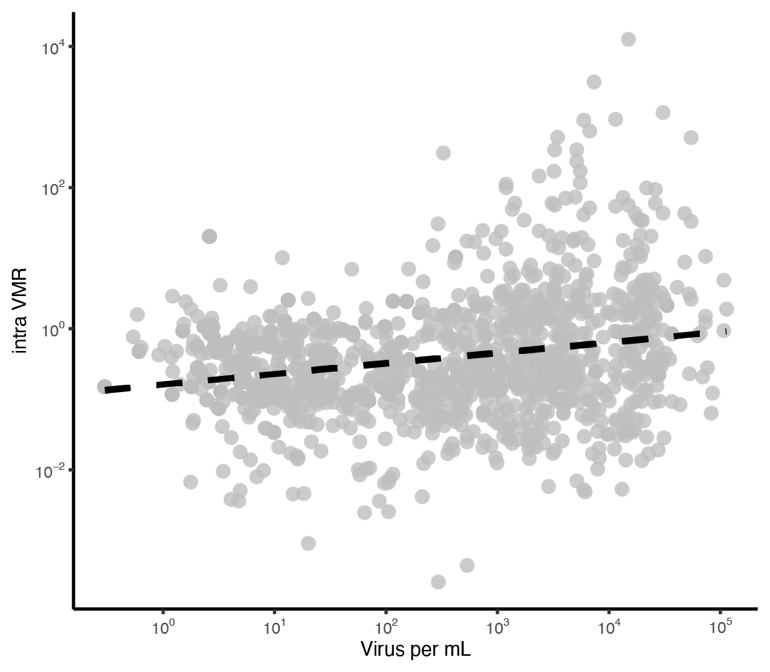
**

**Supplementary Figure 10.** **Relationship between cell-associated VHR and viral abundance in genomes/ml.** Linear regression, P value = < 2e-16, slope= 0.88164 R^2^= 0.08.

**Supplementary Table Legends**

**Supplementary Table 1.** Summary of sampling sites, including location name, geographical coordinates, time and depth of sampling, seawater temperature, and microbial abundances determined by epifluorescence microscopy. **Supplementary Table 2.** Summary of sequencing statistics for viromes (< 0.45 μm) and metagenomes (> 0.22 μm), including total number of reads, quality, and number of reads removed at each step of quality control. **Supplementary Table 3.** Summary of sequencing statistics for Hi-C metagenomes. **Supplementary Table 4.** Pre-dereplication viral contigs and vMAGs and their species-level cluster (population). **Supplementary Table 5.** Viral genomes identified in bacterial contigs as prophages, including the alignment length, identity, bacterial flanking regions and hi-c linkages. **Supplementary Table 6.** CRISPR spacer matching between bMAGs and vMAGs or viral contigs, including alignment length and identity. **Supplementary Table 7.** Host prediction for viral genomes with matches to CRISPR spacers in unassembled reads. **Supplementary Table 8.** List of prokaryotic MAGs, their rank, relative abundances, abundances in genomes/ml, viral links, and taxonomy. **Supplementary Table 9.** Sample-specific virus-host link, indicating the viral genome ID, the host genome ID, viral genome length, viral relative abundance, viral abundances in genomes/ml, type of host link, host genome completeness. **Supplementary Table 10.** List of identified links, indicating the viral genome (contig or vMAG) linked, host, and link type. **Supplementary Table 11.** Metabolic genes identified in viral genomes. **Supplementary Table 12.** Metabolic genes identified in bMAGs. abundance, and taxonomy. **Supplementary Table 13.** Host predictions for the ten most abundant viral genomes in the dataset.
